# Cell-free protein synthesis energized by slowly-metabolized maltodextrin

**DOI:** 10.1186/1472-6750-9-58

**Published:** 2009-06-28

**Authors:** Yiran Wang, Y-H Percival Zhang

**Affiliations:** 1 Biological Systems Engineering Department, Virginia Polytechnic Institute and State University, Blacksburg, Virginia 24061, USA; 2 Institute for Critical Technology and Applied Science (ICTAS), Virginia Polytechnic Institute and State University, Blacksburg, Virginia 24061, USA

## Abstract

**Background:**

Cell-free protein synthesis (CFPS) is a rapid and high throughput technology for obtaining proteins from their genes. The primary energy source ATP is regenerated from the secondary energy source through substrate phosphorylation in CFPS.

**Results:**

Distinct from common secondary energy sources (e.g., phosphoenolpyruvate – PEP, glucose-6-phosphate), maltodextrin was used for energizing CFPS through substrate phosphorylation and the glycolytic pathway because (i) maltodextrin can be slowly catabolized by maltodextrin phosphorylase for continuous ATP regeneration, (ii) maltodextrin phosphorylation can recycle one phosphate per reaction for glucose-1-phosphate generation, and (iii) the maltodextrin chain-shortening reaction can produce one ATP per glucose equivalent more than glucose can. Three model proteins, esterase 2 from *Alicyclobacillus acidocaldarius*, green fluorescent protein, and xylose reductase from *Neurospora crassa *were synthesized for demonstration.

**Conclusion:**

Slowly-metabolized maltodextrin as a low-cost secondary energy compound for CFPS produced higher levels of proteins than PEP, glucose, and glucose-6-phospahte. The enhancement of protein synthesis was largely attributed to better-controlled phosphate levels (recycling of inorganic phosphate) and a more homeostatic reaction environment.

## Background

Cell-free protein synthesis (CFPS) is widely used for the production of *in vivo *cytotoxic, regulatory, or unstable proteins that are difficult to express in living cells [1-3] and for the incorporation of unnatural amino acids into protein polypeptides [4-6]. Also, CFPS can be applied for high-throughput screening of target proteins [7,8], functional tool for proteomics [7,9], and the development of point-of-care medicines [10,11]. Also, CFPS is the fastest method to produce a desired protein within one day [9,12].

Protein synthesis is an energy-intensive process. The primary energy source ATP is regenerated from the secondary energy source through substrate phosphorylation in CFPS [13]. Common secondary energy compounds such as creatine phosphate, phosphoenolpyruvate (PEP), acetate phosphate, glucose-6-phosphate (G6P), 3-phosphoglycerate, and fructose-1,6-biphosphate, contain high-energy phosphate bonds for regenerating ATP. Consequently, ATP regeneration accompanied with the consumption of phosphate-containing substrate leads to an increase in inorganic phosphate. High concentrations of inorganic phosphate severely impair protein biosynthesis, likely due to precipitation of free magnesium ions [14,15]. Magnesium ions are essentially important for nucleoside triphosphate synthesis, protein translation, and translation termination [16,17].

**Figure 1:**
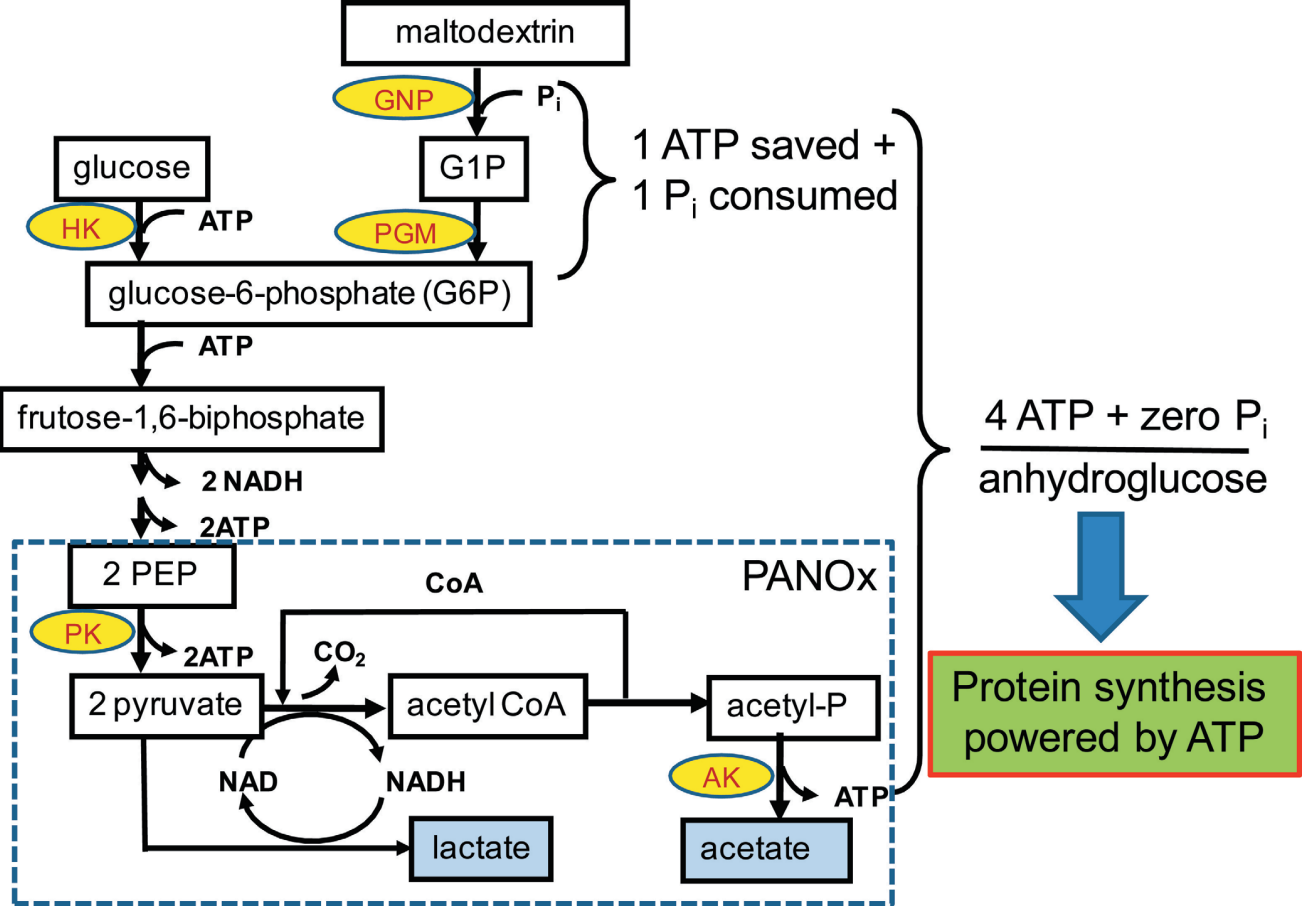
**Pathway of maltodextrin phosphorolysis and glycolysis for regeneration of ATP and recycling of inorganic phosphate**. Maltodextrin phosphorylase, MP; phosphoglucomutase, PGM; and hexokinase, HK.

In order to mitigate the accumulation of free phosphate, Swartz and his coworkers have studied the replacement of costly phosphate-containing secondary energy compounds by using phosphate-free secondary energy compounds (e.g., glucose or pyruvate) [18,19]. Pyruvate, an end product of glycolysis, coupled with the *Pediococcus *sp. pyruvate oxidase, catalase, and acetate kinase to produce one ATP per pyruvate without accumulation of phosphate can be used for long-time CFPS [19]. Further improvements in utilizing glucose through the glycolytic pathway to acetate and lactate have been achieved for more efficient ATP regeneration [14,18,20]. Figure 1 shows that glucose and G6P can produce two and three ATP, respectively, through glycolysis to pyruvate. Extra ATP can be produced through the PANOx system with addition of nicotinamide adenine dinucleotide (NAD^+^) and coenzyme A (CoA) that help convert pyruvate to acetate and lactate. In addition, glucose as the secondary energy source is much less expensive than any phosphate-containing compounds or phosphate-free pyruvate [18].

In addition to the effects of phosphate and the costs of a secondary energy source, stable pH is vital for high protein yields [18,21]. It is well known that well-controlled continuous CFPS systems have high protein yields [3,22]. But continuous CFPS systems with complicated configurations are hard to scale up for high-throughput applications and are not efficient for utilizing costly reagents [21]. It would be appealing to use slowly-metabolized secondary energy sources in batch reactions, which likely mimic the well-controlled continuous reactions.

In this study, to avoid phosphate accumulation and to maintain a relatively homeostatic reaction environment for CFPS, we designed a novel ATP regeneration system starting from low-cost maltodextrin and ending with acetate and lactate, by using a combination of maltodextrin phosphorylase, phosphoglucomutase, glycolysis, and the PNAOx system.

## Results

### A new ATP regeneration pathway

The ATP regeneration scheme was designed to involve maltodextrin phosphorolysis, glycolysis, and PANOx pathway (Fig. 1). Maltodextrin was slowly cleaved into glucose-1-phosphate (G1P) in the presence of inorganic phosphate catalyzed by maltodextrin phosphorylase (Eq. 1). G1P was further converted into G6P by phosphoglucomutase (Eq. 2). The G6P generation rate from maltodextrin can be controlled by the amounts of the added maltodextrin phosphorylase and phosphoglucomutase. We tested the effects of maltodextrin phosphorylase and phosphoglucomutase loading on the EST2 yields. Without addition of maltodextrin phosphorylase and phosphoglucomutase, the EST2 expression levels by *E. coli *cell extract plus maltodextrin were very low (data not shown). The optimal enzyme additions were found to be 0.009 U of maltodextrin phosphorylase and 0.06 U of phosphoglucomutase per 30 μL of the reaction solution. Three moles of ATP can be generated per mole of G6P through the glycolytic pathway (Eq. 3). The PANOx system can convert two molecules of pyruvate to lactate and acetate plus one ATP (Eq. 4).

(1)

(2)

(3)

(4)

The overall pathway as designed can generate four ATP per glucose equivalent of maltodextrin (Eq. 5).

(5)

### Synthesis of esterase

**Figure 2:**
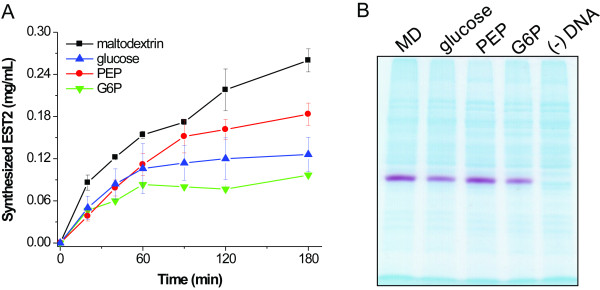
**The synthesized EST2 energized by maltodextrin (MD), glucose, G6P, and PEP**. (A) Profile of the EST2 synthesis levels based on its activity assays. The data were measured in triplicate. (B) Images of SDS-PAGE with stained esterase activity (magenta bands) for cell-free protein synthesis after incubation for 3 hours.

Esterase 2 (EST2) from *Alicyclobacillus acidocaldorius*, a 34 kDa protein, was chosen as a reporter protein for *in vitro *protein synthesis [4,23,24]. Figure 2A shows the profiles of the synthesized active protein EST2 energized by maltodextrin, glucose, PEP, and G6P, respectively. The reaction energized by maltodextrin had the highest protein level, at 0.26 mg EST2 per mL, higher than the other compounds (0.18 mg EST2 per mL, PEP; 0.13 mg EST2 per mL, glucose; 0.09 mg EST2 per mL, G6P). EST2 activity staining on polyacrylamide gel was used to analyze the synthesized proteins after SDS-PAGE separation (Fig. 2B) [25]. The EST2 activity staining helps locating EST2 from a high background of other proteins [26]. The quantitative results based on the EST2 enzyme activity suggested that using maltodextrin as a secondary energy source for cell-free protein synthesis produced more EST2 than the other three energy sources.

**Figure 3:**
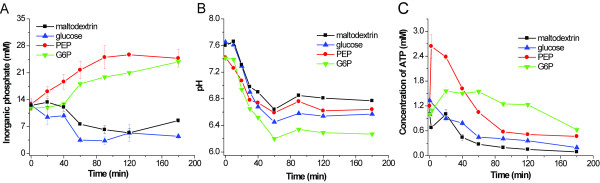
**Profile of inorganic phosphate, pH and ATP levels**. (A) inorganic phosphate concentration, (B) pH, and (C) ATP levels in CFPS energized by maltodextrin, glucose, G6P, and PEP, respectively. The data were measured in triplicate.

Figure 3A presents the profile of inorganic phosphate concentration during CFPS. In the cases of phosphate-free secondary energy compounds (maltodextrin and glucose), inorganic phosphate concentration was nearly constant or even decreased, due to substrate phosphorylation, which consumes free phosphate. In contrast, in the cases of phosphate-containing compounds (PEP and G6P), phosphate levels rose to approximately 26 mM and then levelled off. These increases in phosphate were mainly due to the non-productive hydrolysis of PEP and G6P by the endogenous phosphatases of the *E. coli *cell extract. Phosphate recycling by maltodextrin consumption was efficient for avoiding any possible accumulation of phosphate, resulting in high protein yields.

Changes in pH were measured during the protein synthesis process (Fig. 3B). At the beginning, the initial pHs for the four reactions were around pH 7.4–7.6. The pH from the reactions energized by maltodextrin, PEP, glucose and G6P decreased slowly to 6.9, 6.6, 6.5 and 6.2 after one hour, respectively, and then levelled off. The reaction energized by maltodextrin exhibited the least pH fluctuation, from 7.6 to 6.9.

Concentrations of ATP in the reaction mixtures were measured during the process of protein synthesis (Fig. 3C). In the case of maltodextrin, the ATP level decreased slowly; its levels remained above 0.15 mM for the first two hours. In the case of PEP, the ATP level was highest at the beginning, quickly decreased from 2.67 mM to 0.58 mM, and then levelled off. PEP was an easily metabolized substrate, resulting in higher levels of ATP as compared to other slowly-metabolized substrates of maltodextrin, G6P, and glucose. The measured ATP concentration was the combinatorial result of continuous *in situ *ATP regeneration and ATP consumption for protein synthesis and nonproductive hydrolysis. Recently, it was found that CFPS had a low ATP binding constant (i.e., K_m_^ATP ^= 27 uM) [27], suggesting that ATP levels in CFPS (Fig. 3C) were saturated, *i.e*., the protein synthesis rates did not depend on the ATP levels [19,21].

### Synthesis of CBM-GFP and xylose reductase

**Figure 4:**
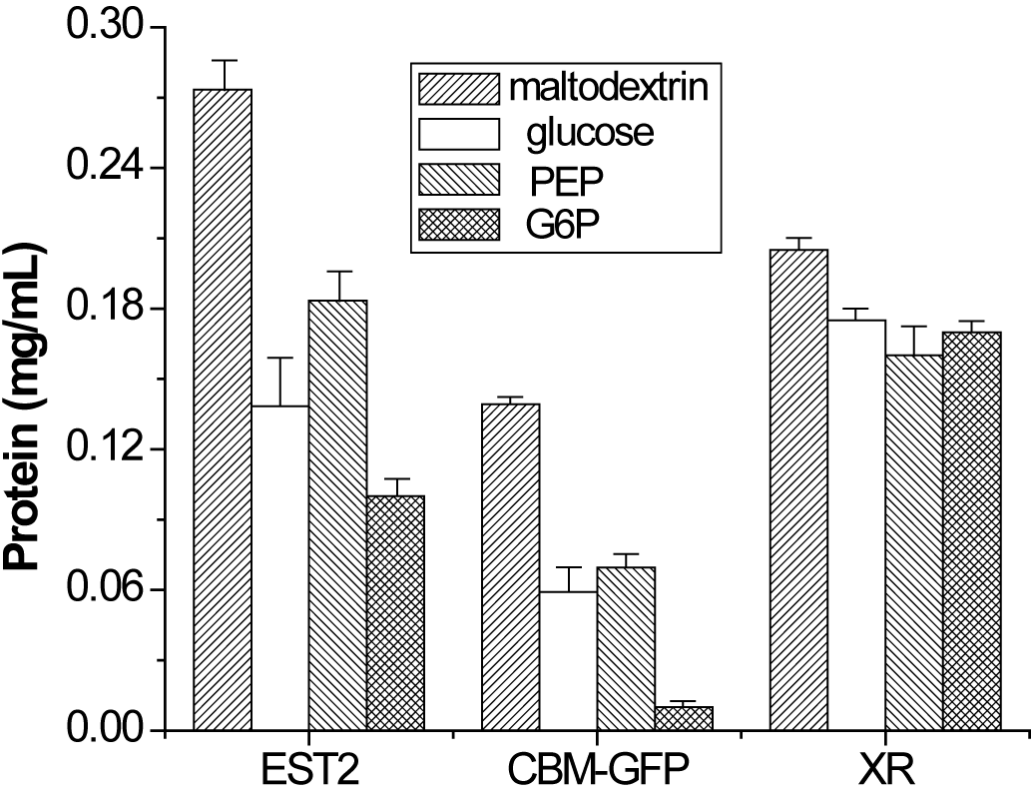
**The CFPS for active EST2, active CBM-GFP, and purified xylose reductase by using different secondary energy compounds**. The data were measured in triplicate.

Two other proteins (CBM-GFP and xylose reductase) were investigated for CFPS energized by maltodextrin, glucose, PEP, and G6P, respectively. Figure 4 shows that the final CBM-GFP and xylose reductase concentrations are 0.14 and 0.21 mg per mL, respectively, when maltodextrin was used as secondary energy source. These values were higher than those powered by PEP, G6P and glucose but the comparative advantages depended on the protein type. Slowly metabolized, low-cost maltodextrin may be a good secondary energy source for CFPS without the need for intensive optimization.

## Discussion

CFPS energized by maltodextrin presented relatively high levels of EST2, CBM-GFP and xylose reductase as compared with three other secondary energy sources – glucose, G6P, and PEP. Maltodextrin provided relatively homeostatic conditions, such as no phosphate accumulation and less fluctuation in pH (Fig. 3A &3B). Slowly metabolized maltodextrin in batch CFPS was somewhat like continuous CFPS with regard to well-controlled reaction conditions.

The major advantages of using slowly-metabolized maltodextrin are to avoid inorganic phosphate accumulation to inhibitory levels and to maintain a relatively homeostatic environment (e.g., phosphate, pH, and ATP) for protein synthesis. Proper levels of inorganic phosphate are also required for effective synthesis of nucleoside triphosphate (NTP) from nucleoside diphosphate (NDP) for high level protein synthesis [18,19]. Using phosphate-free maltodextrin does not release net phosphate. In contrast, it can decrease free phosphate levels by substrate phosphorylation and keep phosphate levels nearly constant. In addition, maltodextrin is not a substrate of *E. coli *endogenous phosphatases. Therefore, the challenge associated with non-productive hydrolysis of phosphate-containing compounds [19,28] has been partially addressed by using maltodextrin.

The importance of pH control in CFPS has long been recognized [18,21]. More stable pH in the case of maltodextrin was largely attributed to no phosphate accumulation and less formation of organic acids (e.g., lactate and acetate) due to the generation of more ATP per substrate consumed (Additional file 1). The latter speculation has been validated by product assays, in which 40, 18 and 18 mM lactate were produced from glucose, maltodextrin and PEP, respectively, after a 1 hour reaction. Swartz and his coworkers also reported that a strong pH-capacity buffer (Bis-Tris, pH 6.5) increased protein synthesis levels [18].

EST2 was used as a major model protein for CFPS in this study. Due to its high-sensitivity and thermal stability, it had been applied not only for CFPS [4,23,24] but also for detecting nucleic acid hybridization [29,30]. Since EST2 has a specific activity of 1100 U/mg based on *p*-nitrophenylbutyrate at 25°C [31], its levels can be measured as low as 72 pM, superior to ^14^C radioactivity-labelled amino acids. We also used the Roche High-Yield *in vitro *protein synthesis system as a control to express EST2 protein at a level of 0.21 mg per mL. The previous EST2 levels by CFPS were 0.15–0.20 mg per mL [24,29]. The expression level (0.28 mg of EST2 per mL) energized by maltodextrin was the highest so far.

A number of secondary energy substrates were compared based on their ATP yields and phosphate release (Additional file 1). Acetyl-phosphate, creatine-phosphate, 3-phosphoglycerate (3-PGA), and PEP generated one ATP and released one inorganic phosphate [13,32-34]. ATP regeneration potential through the PANOx system was approximately one ATP per two pyruvate with an acetate/lactate = ~1:1. In principle, one molecule of glucose, G6P and fructose-1, 6-biphosphate can regenerate three, four and five molecules of ATP and release zero, one and two phosphate, respectively. One glucose equivalent of maltodextrin can produce four molecules of ATP, while it does not release phosphate (Eq. 5). Maltodextrin is better than glucose because generation of G1P mediated by glucan phosphorylases from polysaccharides does not consume ATP so that one molecule of ATP is saved from glucose phosphorylation catalyzed by ATP-dependent hexokinase [35-37]. It is well known that ATP consumption of CFPS is not coupled to ATP regeneration [18,20], which is different from *in vivo *protein synthesis [38,39]. But slowly-metabolized maltodextrin can enhance the protein synthesis yield and decrease energy dissipation because they can avoid an initial high concentration of the secondary energy compound, most of which was hydrolyzed by *E. coli *endogenous phosphatases. For example, approximately 70% of 30 mM PEP was hydrolyzed within the first 30 min by endogenous phosphatase in the *E. coli *cell extract [19].

In addition to the high product levels associated with more homeostatic conditions, maltodextrin is a low-cost substrate. The secondary energy compound cost accounts for a significant fraction of total CFPS cost [18,40]. For example, PEP costs $1.20 per mL of reaction mixture (30 mM PEP), while glucose and maltodextrin cost only $0.0002 and $0.001 per mL of reaction mixture, respectively, based on Sigma-Aldrich prices. Although a small amount of maltodextrin phosphorylase (0.009 U per reaction) and phosphoglucomutase (0.06 U per reaction) added some extra costs for the entire systems, two over-expressed recombinant enzymes in *E. coli *were produced and used in this study [41,42]. It is anticipated that costs of supplementary enzymes will be decreased to very low levels. The use of maltodextrin in CFPS can not only dramatically decrease the cost of secondary energy but also increase synthesized protein levels.

## Conclusion

The use of maltodextrin as a secondary energy source for cell-free protein synthesis was shown to synthesize proteins with several advantages: relatively high protein synthesis, no phosphate accumulation, little change in pH, and low cost. The slowly generated G1P from maltodextrin followed by glycolysis provided relatively homeostatic CFPS environments.

## Methods

### Materials

A total tRNA mixture from *E. coli *MRE600 and the *in vitro *protein synthesis system (RTS 100 *E. coli *High-yield Kit) were obtained from Roche Diagnostics (Indianapolis, IN, USA). The 20 amino-acid mixture was purchased from Spectra Stable Isotopes (Columbia, MD, USA). The recombinant α-glucan phosphorylase and phosphoglucomutase from *Clostridium thermocellum *were prepared as described elsewhere [41,42]. The plasmid pIVEX2.3d-est2 encoding the esterase 2 (*est2*) from *Alicyclobacillus acidocaldarius *was described elsewhere [24]. The plasmid pET26b-XR encoding xylose reductase from *Neurospora crassa *was described elsewhere [43]. The plasmid pCG encoding cellulose-binding module and green fluorescent protein (CBM-GFP) was described previously [44]. *Pfx*50 DNA polymerase and Ni-NTA agarose were purchased from Invitrogen Co. (Carlsbad, CA, USA). *E. coli *Bl21(DE3) was purchased from Novagen (Gibbstown, NJ, USA). The rest of the reagents were purchased from Sigma-Aldrich (St. Louis, MO, USA).

### Construction of the expression plasmids

The plasmids of pIVEX2.3d-xr for xylose reductase and pIVEX2.3d-cg for CBM-GFP were constructed based on the plasmid pIVEX2.3d-est2. The DNA fragment of xylose reductase gene was amplified by the primers Xr-F (5'-GACTGACT**CATATG**GTTCCTGCTATCAAGCTCAAC-3') and Xr-R (5'-GACTGACT**TGATCA**TCAGTGGTGGTGGTGGTGG-3') on the DNA template of pET26b-XR (bolded nucleotide sequences are NdeI and BclI sites). The plasmid pIVEX2.3d-xr was constructed after NdeI- and BclI-digestion of PCR product and ligation with the NdeI- and BamHI-digested plasmid pIVEX2.3d-est2. The plasmid pIVEX2.3d-cg for CBM-GFP was constructed by ligation of the NdeI- and BamHI-digested CBM-GFP DNA fragment from the plasmid pCG and ligation with the NdeI- and BamHI-digested pIVEX2.3d-est2. The plasmids were validated by DNA sequencing.

### Preparation of the *E. coli *S12 extract

The *E. coli *S12 extracts were prepared from *E. coli *BL21(DE3) according to the Kim protocol [45]. The strain was grown in 4 litres of 2xYT medium in a 6.6-L NBS BioFlo 310 Bioreactor (Edison, NJ). The cultivation conditions were 37°C, 200 rpm, and 1.0 vvm. When the absorbance (A_600_) reached 0.6, a final concentration of 1 mM IPTG was added, and then agitation and airflow rates were increased to 300 rpm and 2.0 vvm respectively. The cells were harvested in the middle exponential phase (A_600 _= ~4.0). After centrifugation, the cell pellets were washed by an ice-cooled 10 mM Tris-acetate buffer (pH 8.2) with 60 mM potassium glutamate, 14 mM magnesium acetate, 1 mM DTT, and 0.05% beta-mercaptoethnaol three times and then were stored in -80°C. The cell extract was prepared by French Press at a pressure of 20,000 psi. After centrifugation at 12,000 relative centrifugal force (RCF) for 10 min, the supernatant was briefly incubated at 37°C in 10 mM Tris-acetate buffer (pH 8.2) with 60 mM potassium glutamate, 14 mM magnesium acetate, 1 mM DTT for 10 min and then was stored at -80°C for future cell-free protein synthesis. The S12 extracts had 33 mg of cellular protein per mL.

### Cell-free protein biosynthesis

The CFPS mixture (30 μL, final volume) consisted of 57 mM of Hepes-KOH (pH 8.2), 1.2 mM of ATP, 0.85 mM of CTP, GTP and UTP (each), 0.6 mM cAMP, 2 mM of DTT, 0.17 mg per mL of *E. coli *MRE600 total tRNA mixture, 90 mM of potassium glutamate, 80 mM of ammonium acetate, 12 mM of magnesium acetate, 34 μg per mL of l-5-formyl-5,6,7,8-tetrahydrofolic acid, 1.5 mM each of 20 amino acids, 2% (w/v) of PEG 8000, 10 U per mL of pyruvate kinase, 5 nM template DNA, 0.33 mM NAD, 0.26 mM CoA, and 27% (v/v) of the S12 extract. The secondary energy compound (30 mM) was either PEP, glucose, G6P, or maltodextrin. CFPS was conducted at 37°C. When maltodextrin was used as a secondary energy source, the reaction solution was supplemented with 0.009 U of the recombinant *C. thermocellum *α-glucan phosphorylase and 0.06 U of phosphoglucomutase to generate glucose-1-phosphate (G1P) from maltodextrin for energizing CFPS.

### Esterase activity staining on polyacrylamide gel

The protein esterase 2 in the CFPS solution was separated by SDS-polyacrylamide gel in a 100 mM Tris-HCl (pH 7.5). After electrophoresis and EST2 renaturation by removing SDS, the gel was mixed with the substrate (β-naphthylacetate) and the product (β-naphthol) was linked with Fast Blue BB for the formation of an insoluble color complex, indicating the presence and amount of EST2 [24,46]. Subsequently, the other proteins in the gels were strained by the Coomassie blue dye [47].

### Protein assays

Molar concentration of EST2 (mol/L) was determined by the ratio of the spectrophotometric assay of esterase activity to its molar-specific activity according to [EST2] = (A_405_/min-0.016)/6.10 × 10^8^, where A_405_/min is absorbance of a 1-cm path length at 405 nm per minute for a EST2 concentration ranging from 0.02 to 1 nM [31]. Two μL of the reaction mixture were withdrawn and diluted 100-fold in an assay buffer (30 mM Tris-HCl, pH 7.5, 200 mM NaCl and 0.05% Triton X-100). Either 2 μL of the diluted EST2 solution or a further 10-fold diluted EST2 solution was mixed to a final 250 μL of the assay buffer containing 0.2 mM *p*-nitrophenylbutyrate. The synthesized CBM-GFP protein was quantified in a 50 mM Hepes solution (pH 7.0) using a BioTek multi-detection microplate reader with a reference of purified CBM-GFP expressed by *E. coli *[44,48]. The synthesized XR protein was determined by Ni-NTA adsorption and desorption followed by the Bio-Rad Bradford assay.

### Other assays

Concentration of inorganic phosphate in the reaction mixture was determined by using a mild-pH phosphate assay as described elsewhere [49]. Concentration of ATP was measured by the Promega ATP assay kit (Madison, WI) based on the known concentration of ATP standards, as described elsewhere [20] with modifications. A 2 μL reaction mixture was diluted by 100-fold in the EST2 activity assay buffer, and then 50 μL diluted solution was added into 450 μL of water that had been boiled to denature ATP hydrolysis enzymes, i.e. ATPase and phosphatases [50]. The diluted solution stood on ice until the ATP assay. The pH of the CFPS solution was measured by using an Orion 9810BN micro-pH probe (Beverly, MA) at 37°C. Concentration of lactate was measured with a Beckman-Coulter HPLC (Fullerton, CA) equipped with the Bio-Rad HPX-87 column as described elsewhere [51].

## Authors' contributions

YW and YHPZ designed the experiment. YW performed the experiments. YW and YHPZ wrote the manuscript. Both authors read and approved the final manuscript.

## Supplementary Material

Additional file 1 **Comparison of ATP generation approaches using secondary energy compounds for cell-free protein synthesis**. The amount of ATP generated, the involved enzymes and pathways for ATP generation, the accompanied inorganic phosphate upon the consumption of different secondary energy compounds for cell-free protein synthesis was summarized. Click here for file

## References

[B1] Stiege W, Erdmann VA (1995). The potentials of the *in vitro *protein biosynthesis system. J Biotechnol.

[B2] Boyer ME, Stapleton JA, Kuchenreuther JM, Wang CW, Swartz JR (2008). Cell-free synthesis and maturation of [FeFe] hydrogenases. Biotechnol Bioeng.

[B3] Spirin AS, Baranov VI, Ryabova LA, Ovodov SY, Alakhov YB (1988). A continuous cell-free translation system capable of producing polypeptides in high yield. Science.

[B4] Agafonov DE, Rabe KS, Grote M, Voertler CS, Sprinzl M (2006). C-terminal modifications of a protein by UAG-encoded incorporation of puromycin during *in vitro *protein synthesis in the absence of release factor 1. ChemBioChem.

[B5] Hirao I, Ohtsuki T, Fujiwara T, Mitsui T, Yokogawa T, Okuni T, Nakayama H, Takio K, Yabuki T, Kigawa T (2002). An unnatural base pair for incorporating amino acid analogs into proteins. Nat Biotechnol.

[B6] Noren CJ, Anthony-Cahill SJ, Griffith MC, Schultz PG (1989). A general method for site-specific incorporation of unnatural amino acids into proteins. Science.

[B7] He M, Taussig MJ (2007). Rapid discovery of protein interactions by cell-free protein technologies. Biochem Soc Trans.

[B8] Lamla T, Erdmann VA (2003). Searching sequence space for high-affinity binding peptides using ribosome display. J Mol Biol.

[B9] Sawasaki T, Ogasawara T, Morishita R, Endo Y (2002). A cell-free protein synthesis system for high-throughput proteomics. Proc Natl Acad Sci USA.

[B10] He M, Khan F (2005). Ribosome display: next-generation display technologies for production of antibodies *in vitro*. Exp Rev Proteomics.

[B11] Yang J, Kanter G, Voloshin A, Michel-Reydellet N, Velkeen H, Levy R, Swartz JR (2005). Rapid expression of vaccine proteins for B-cell lymphoma in a cell-free system. Biotechnol Bioeng.

[B12] Lesley SA, Brow MA, Burgess RR (1991). Use of *in vitro *protein synthesis from polymerase chain reaction-generated templates to study interaction of *Escherichia coli *transcription factors with core RNA polymerase and for epitope mapping of monoclonal antibodies. J Biol Chem.

[B13] Zubay G (1973). *In vitro *synthesis of protein in microbial systems. Annu Rev Genet.

[B14] Jewett MC, Swartz JR (2004). Rapid expression and purification of 100 nmol quantities of active protein using cell-free protein synthesis. Biotechnol Prog.

[B15] Kim DM, Choi CY (1996). A semicontinuous prokaryotic coupled transcription/translation system using a dialysis membrane. Biotechnol Prog.

[B16] Levit MN, Abramczyk BM, Stock JB, Postel EH (2002). Interactions between *Escherichia coli *nucleoside-diphosphate kinase and DNA. J Biol Chem.

[B17] Sprinzl M (1994). Elongation factor Tu: a regulatory GTPase with an integrated effector. Trends Biochem Sci.

[B18] Calhoun KA, Swartz JR (2005). Energizing cell-free protein synthesis with glucose metabolism. Biotechnol Bioeng.

[B19] Kim DM, Swartz JR (1999). Prolonging cell-free protein synthesis with a novel ATP regeneration system. Biotechnol Bioeng.

[B20] Kim DM, Swartz JR (2001). Regeneration of adenosine triphosphate from glycolytic intermediates for cell-free protein synthesis. Biotechnol Bioeng.

[B21] Jewett MC, Swartz JR (2004). Mimicking the *Escherichia coli *cytoplasmic environment activates long-lived and efficient cell-free protein synthesis. Biotechnol Bioeng.

[B22] Kigawa T, Yabuki T, Yoshida Y, Tsutsui M, Ito Y, Shibata T, Yokoyama S (1999). Cell-free production and stable-isotope labeling of milligram quantities of proteins. FEBS Lett.

[B23] Agafonov DE, Huang Y, Grote M, Sprinzl M (2005). Efficient suppression of the amber codon in *E. coli in vitro *translation system. FEBS Lett.

[B24] Agafonov DE, Rabe KS, Grote M, Huang Y, Sprinzl M (2005). The esterase from *Alicyclobacillus acidocaldarius *as a reporter enzyme and affinity tag for protein biosynthesis. FEBS Lett.

[B25] Koch M, Huang Y, Sprinzl M (2008). Peptide-bond synthesis on the ribosome: no free vicinal hydroxy group required on the terminal ribose residue of peptidyl-tRNA. Angew Chem Int Ed.

[B26] Jungbauer LM, Cavagnero S (2006). Characterization of protein expression and folding in cell-free systems by maldi-tof mass spectrometry. Anal Chem.

[B27] Jewett MC, Miller ML, Chen Y, Swartz JR (2009). Continued protein synthesis at low [ATP] and [GTP] enables cell adaptation during energy limitation. J Bacteriol.

[B28] Kawarasaki Y, Nakano H, Yamane T (1998). Phosphatase-immunodepleted cell-free protein synthesis system. J Biotechnol.

[B29] Humenik M, Huang Y, Wang Y, Sprinzl M (2007). C-terminal incorporation of bio-orthogonal azide groups into a protein and preparation of protein-oligodeoxynucleotide conjugates by Cu'-catalyzed cycloaddition. ChemBioChem.

[B30] Wang Y, Stanzel M, Gumbrecht W, Humenik M, Sprinzl M (2007). Esterase 2-oligodeoxynucleotide conjugates as sensitive reporter for electrochemical detection of nucleic acid hybridization. Biosens Bioelectron.

[B31] Wang Y (2007). Esterase 2-oligodeoxynucleotide conjugates as enzyme reporter for electrochemical detection of DNA and identification of bacterial species. PhD Thesis.

[B32] Ryabova LA, Vinokurov LM, Shekhovtsova EA, Alakhov YB, Spirin AS (1995). Acetyl phosphate as an energy source for bacterial cell-free translation systems. Anal Biochem.

[B33] Weber LA, Feman ER, Baglioni C (1975). A cell free system from HeLa cells active in initiation of protein synthesis. Biochemistry.

[B34] Sitaraman K, Esposito D, Klarmann G, Le Grice SF, Hartley JL, Chatterjee DK (2004). A novel cell-free protein synthesis system. J Biotechnol.

[B35] Ye X, Wang Y, Hopkins RC, Adams MWW, Evans BR, Mielenz JR, Zhang Y-HP (2009). Spontaneous high-yield production of hydrogen from cellulosic materials and water catalyzed by enzyme cocktails. ChemSusChem.

[B36] Zhang Y-HP, Evans BR, Mielenz JR, Hopkins RC, Adams MW (2007). High-yield hydrogen production from starch and water by a synthetic enzymatic pathway. PLoS ONE.

[B37] Zhang Y-HP, Lynd LR (2004). Kinetics and relative importance of phosphorolytic and hydrolytic cleavage of cellodextrins and cellobiose in cell extracts of *Clostridium thermocellum*. Appl Environ Microbiol.

[B38] Thauer RK, Jungermann K, Decker K (1977). Energy conservation in chemotrophic anaerobic bacteria. Bacteriol Rev.

[B39] Zhang Y-HP, Lynd LR (2005). Cellulose utilization by *Clostridium thermocellum*: bioenergetics and hydrolysis product assimilation. Proc Natl Acad Sci USA.

[B40] Kim TW, Keum JW, Oh IS, Choi CY, Kim HC, Kim DM (2007). An economical and highly productive cell-free protein synthesis system utilizing fructose-1,6-bisphosphate as an energy source. J Biotechnol.

[B41] Hong J, Wang Y, Ye X, Zhang Y-HP (2008). Simple protein purification through affinity adsorption on regenerated amorphous cellulose followed by intein self-cleavage. J Chromatogr A.

[B42] Wang Y, Zhang Y-HP (2009). A highly active phosphoglucomutase from *Clostridium thermocellum*: Cloning, purification, characterization, and enhanced thermostability. J Appl Microbiol.

[B43] Woodyer R, Simurdiak M, Donk WA van der, Zhao H (2005). Heterologous expression, purification, and characterization of a highly active xylose reductase from *Neurospora crassa*. Appl Environ Microbiol.

[B44] Hong J, Ye X, Wang Y, Zhang Y-HP (2008). Bioseparation of recombinant cellulose-binding module-proteins by affinity adsorption on an ultra-high-capacity cellulosic adsorbent. Anal Chim Acta.

[B45] Kim TW, Keum JW, Oh IS, Choi CY, Park CG, Kim DM (2006). Simple procedures for the construction of a robust and cost-effective cell-free protein synthesis system. J Biotechnol.

[B46] Higerd TB, Spizizen J (1973). Isolation of two acetyl esterases from extracts of *Bacillus subtilis*. J Bacteriol.

[B47] Zhang Y-HP, Lynd LR (2003). Quantification of cell and cellulase mass concentrations during anaerobic cellulose fermentation: development of an enzyme-linked immunosorbent assay-based method with application to *Clostridium thermocellum *batch cultures. Anal Chem.

[B48] Hong J, Ye X, Zhang Y-HP (2007). Quantitative determination of cellulose accessibility to cellulase based on adsorption of a nonhydrolytic fusion protein containing CBM and GFP with its applications. Langmuir.

[B49] Saheki S, Takeda A, Shimazu T (1985). Assay of inorganic phosphate in the mild pH range, suitable for measurement of glycogen phosphorylase activity. Anal Biochem.

[B50] Yang NC, Ho WM, Chen YH, Hu ML (2002). A convenient one-step extraction of cellular ATP using boiling water for the luciferin-luciferase assay of ATP. Anal Biochem.

[B51] Zhang Y-HP, Lynd LR (2005). Regulation of cellulase synthesis in batch and continuous cultures of *Clostridium thermocellum*. J Bacteriol.

