# Supplementary material for: Cell-free protein synthesis energized by slowly-metabolized maltodextrin
Source: BMC Biotechnol. 2009 Jun 28;9:58. doi: 10.1186/1472-6750-9-58 (PMC2716334; doi:10.1186/1472-6750-9-58)
Supplement: Additional file 1 — Comparison of ATP generation approaches using secondary energy compounds for cell-free protein synthesis. The amount of ATP generated, the involved enzymes and pathways for ATP generation, the accompanied inorganic phosphate upon the consumption of different secondary energy compounds for cell-free protein synthesis was summarized. [file 1472-6750-9-58-S1.doc]

**Additional file 1.**  Comparison of ATP generation approaches using secondary energy compounds for cell-free protein synthesis.

Substrate^*^ Product Enzyme(s) ATP/Substrate P_i_/Substrate References

Total glycolysis AA

AcP acetate (AA) Acetate kinase (AK) 1 0 1 1 [1]

CrP creatine creatine kinase 1 0 0 1 [2]

PEP pyruvate pyruvate kinase (PK) 1 1 0 1 [3]

3-PGA pyruvate glycolysis 1 1 0 1 [4]

pyruvate acetate pyruvate oxidase + AK 1 0 1 0 [5]

pyruvate AA/lactate PDH + AK/LDH 1 0 1 0 [6]

PEP AA/lactate PK + PDH + AK/LDH 1.5 1 0.5 1 [7]

G6P AA/lactate glycolysis + PDH + AK/LDH 4 3 1 1 [6]

glucose AA/lactate HK+ glycolysis + PDH + AK/LDH 3 2 1 0 [8]

FBP AA/lactate glycolysis + PDH + AK/LDH 5 4 1 2 [9]

maltodextrin AA/lactate GNP/PGN+ glycolysis + PDH + AK/LDH 4 3 1 0 This study

* Abbreviations are acetyl-phosphate (AcP), creatine phosphate (CrP), maltodextrin (MD), fructose-1,6-biphosphate (FBP), pyruvate dehydrogenase (PDH), hexose kinase (HK), inorganic phosphate (P_i_).

1. Ryabova LA, Vinokurov LM, Shekhovtsova EA, Alakhov YB, Spirin AS: **Acetyl phosphate as an energy source for bacterial cell-free translation systems**. *Analytical biochemistry* 1995, **226**(1):184-186.

2. Weber LA, Feman ER, Baglioni C: **A cell free system from HeLa cells active in initiation of protein synthesis**. *Biochemistry* 1975, **14**(24):5315-5321.

3. Zubay G: **In vitro synthesis of protein in microbial systems**. *Annu Rev Genet* 1973, **7**:267-287.

4. Sitaraman K, Esposito D, Klarmann G, Le Grice SF, Hartley JL, Chatterjee DK: **A novel cell-free protein synthesis system**. *J Biotechnol* 2004, **110**(3):257-263.

5. Kim DM, Swartz JR: **Prolonging cell-free protein synthesis with a novel ATP regeneration system**. *Biotechnology and bioengineering* 1999, **66**(3):180-188.

6. Kim DM, Swartz JR: **Regeneration of adenosine triphosphate from glycolytic intermediates for cell-free protein synthesis**. *Biotechnology and bioengineering* 2001, **74**(4):309-316.

7. Jewett MC, Swartz JR: **Rapid expression and purification of 100 nmol quantities of active protein using cell-free protein synthesis**. *Biotechnol Prog* 2004, **20**(1):102-109.

8. Knapp KG, Swartz JR: **Evidence for an additional disulfide reduction pathway in *Escherichia coli***. *J Biosci Bioeng* 2007, **103**(4):373-376.

9. Kim TW, Keum JW, Oh IS, Choi CY, Kim HC, Kim DM: **An economical and highly productive cell-free protein synthesis system utilizing fructose-1,6-bisphosphate as an energy source**. *J Biotechnol* 2007, **130**(4):389-393.
